# Observational Learning of New Movement Sequences Is Reflected in Fronto-Parietal Coherence

**DOI:** 10.1371/journal.pone.0014482

**Published:** 2010-12-31

**Authors:** Jurjen van der Helden, Hein T. van Schie, Christiaan Rombouts

**Affiliations:** 1 Donders Institute for Brain, Cognition and Behaviour, Radboud University, Nijmegen, The Netherlands; 2 Department of Cognitive Psychology and Ergonomics, University of Twente, Enschede, The Netherlands; 3 Behavioural Science Institute, Radboud University Nijmegen, Nijmegen, The Netherlands; University of Alberta, Canada

## Abstract

Mankind is unique in her ability for observational learning, i.e. the transmission of acquired knowledge and behavioral repertoire through observation of others' actions. In the present study we used electrophysiological measures to investigate brain mechanisms of observational learning. Analysis investigated the possible functional coupling between occipital (alpha) and motor (mu) rhythms operating in the 10Hz frequency range for translating “seeing” into “doing”. Subjects observed movement sequences consisting of six consecutive left or right hand button presses directed at one of two target-buttons for subsequent imitation. Each movement sequence was presented four times, intervened by short pause intervals for sequence rehearsal. During a control task subjects observed the same movement sequences without a requirement for subsequent reproduction. Although both alpha and mu rhythms desynchronized during the imitation task relative to the control task, modulations in alpha and mu power were found to be largely independent from each other over time, arguing against a functional coupling of alpha and mu generators during observational learning. This independence was furthermore reflected in the absence of coherence between occipital and motor electrodes overlaying alpha and mu generators. Instead, coherence analysis revealed a pair of symmetric fronto-parietal networks, one over the left and one over the right hemisphere, reflecting stronger coherence during observation of movements than during pauses. Individual differences in fronto-parietal coherence were furthermore found to predict imitation accuracy. The properties of these networks, i.e. their fronto-parietal distribution, their ipsilateral organization and their sensitivity to the observation of movements, match closely with the known properties of the mirror neuron system (MNS) as studied in the macaque brain. These results indicate a functional dissociation between higher order areas for observational learning (i.e. parts of the MNS as reflected in 10Hz coherence measures) and peripheral structures (i.e. lateral occipital gyrus for alpha; central sulcus for mu) that provide low-level support for observation and motor imagery of action sequences.

## Introduction

Many behavioural skills that humans establish during their life are acquired through observational learning. An important neurophysiological principle that has been hypothesized to underlie observational learning and imitation is *motor resonance*, i.e. the automatic activation of motor representations during action observation. Neuroimaging experiments in humans and single cell studies in macaques have investigated the neurophysiological basis of motor resonance during action observation [1]. In particular, motor resonance has been described as a basic property of *mirror neurons*, a selection of motor neurons in monkey higher-order premotor and parietal areas that are activated in a comparable manner during action execution and during the observation of a similar action by another individual [2], [3]. At a larger scale neuroimaging experiments in humans confirm the existence of a mirror neuron system in man comprised of the inferior frontal gyrus, and the inferior parietal lobe supporting both action observation [4] and imitation [5], [6].

The mirror neuron system (MNS) has been shown to be activated to a stronger extent if observers view movements that they already have in their repertoire. Calvo-Merino and others [7], [8] for instance found that the MNS of dancers that observed dancing movements in the fMRI scanner, responded more strongly if these movements were part of their dancing routine. Orgs and others [9] showed that dancing experience was correlated with motor resonance, reflected in mu- and beta-suppression in the dancer's electroencephalogram (EEG), when they observed a familiar dance movement. Recent findings from van Elk and others [10] further extend these findings to the domain of natural motor development. In their study, EEG was recorded from 14- to 16-month old infants during observation of videos showing other infants crawling and walking. EEG mu- and beta-suppression was found strongly related to the infant's own motor experience, suggesting that already early in life an individuals' action experience determines how the actions of others are processed.

Whereas the above studies convincingly showed effects of motor experience to influence the observation of actions by others, involvement of the human MNS in the reverse direction, i.e. during the acquisition of a new action repertoire, has until recently remained untested [11]. Several fMRI studies in the last few years suggest that activity in core areas of the MNS (inferior frontal gyrus and inferior parietal lobe) and in cortical and subcortical regions supporting motor learning and visual perception, increase as a function of the complexity of the movements that are to be acquired [6], [12]–[14]. The results of these experiments are inspiring as they reveal some of the underlying neural circuitry supporting observational learning. However, still little is known about the neural mechanisms and functional processes that operate within the extent of spatial activation patterns as indicated by functional imaging studies. For instance, not much is known about the functional contribution of areas and the integration of information between them in supporting functional aspects of observational learning over time.

Two interesting proposals concerning the possible functional integration of information between areas supporting observational learning have recently been formulated. Iacoboni [5] proposes that observational learning is supported by internal (inverse and forward) models that dynamically link visual and motor processes to support the observation and imitation of new behavior. Internal models were first conceived to serve the execution of actions by allowing sensory structures to anticipate the perceptual consequences of initiated actions (via forward internal models), and use the perceptual error – i.e. the difference between the perceived and anticipated perceptual result – to generate corrective motor commands (using inverse internal models). Iacoboni proposes that similar to action execution, internal models may also be engaged during the observation of others' actions, providing an internal simulation of the observed behavior. In his model, motor representations in parietal and frontal parts of the MNS are dynamically linked with perceptual representations in the posterior superior temporal sulcus. Similarly, Pineda [15] proposes that action observation (e.g. for observational learning) may cause alpha and mu rhythms in respective visual and sensorimotor structures to become transiently linked supporting the translation of “seeing” and/or “hearing” into “doing”. According to this *global entrainment hypothesis* the coupling between perception and action is realized via global alpha/mu entrainment, resulting in widespread coherence between local alpha and mu generators in visual, auditory and motor structures when tasks demand integrated cognitive processing.

However, according to the *direct matching hypothesis* of Rizzolatti, Fogassi, and Gallese [1] imitation learning is primarily associated with resonance in the motor system and has to be distinguished from activation in the visual system. According to this hypothesis observational learning depends on the core areas of the MNS, the inferior frontal gyrus and the inferior parietal lobe that are supporting motor simulation of observed movements. Different from the global entrainment hypothesis, the direct matching hypothesis does not predict alpha and mu source activations to become synchronized during observational learning. Instead, the direct matching hypothesis would predict coupling between frontal and parietal motor regions that make up the core MNS.

Electroencephalograpic (EEG) measurements provide an excellent opportunity to investigate the contribution of visual and motor processes and their hypothesized integration during observational learning. Both EEG power and EEG coherence measures may provide relevant information to uncover the possible relationship between perceptual and action processes in observational learning. Variations in alpha and mu power over time, i.e. during different stages of observational learning, may help to determine a possible relationship between these two components. Also, EEG coherence [16], which provides a measure of the functional connectivity as expressed by the relative phase stability between oscillations recorded from different sites, may be used to uncover relations between different cortical areas supporting observational learning.

Previous EEG studies in the domain of action observation have mostly focused on EEG power and found that observation of a movement is accompanied by a transient drop in the mu and beta power. Mutukumaraswamy and others, for instance [17], [18] have shown the amplitude of the mu rhythm to attenuate when subjects observe a goal-directed grasping movement in much the same way as it is attenuated when subjects performed this same movement themselves. Surprisingly, whereas previous studies have consistently focused on desynchronization of the mu rhythm, modulations in alpha power during action observation have been largely neglected. Similar to the mu rhythm, alpha, which is strongest over visual cortices, is found to desynchronize to the presentation of stimuli (e.g. actions) and during tasks that require visual attention, e.g. for selecting, anticipating or remembering visual stimuli [15], [19]–[22]. Consequently, in previous studies alpha and mu may have been confounded and the possible individual and joint effects of either component to observational learning remain to be determined. Note that although beta power (∼16–30Hz), similar to mu, is found to desynchronize during action observation [9], [23], [33], its sources are mainly centrally distributed and thus not likely to be confounded with a posterior source.

**Figure 1:**
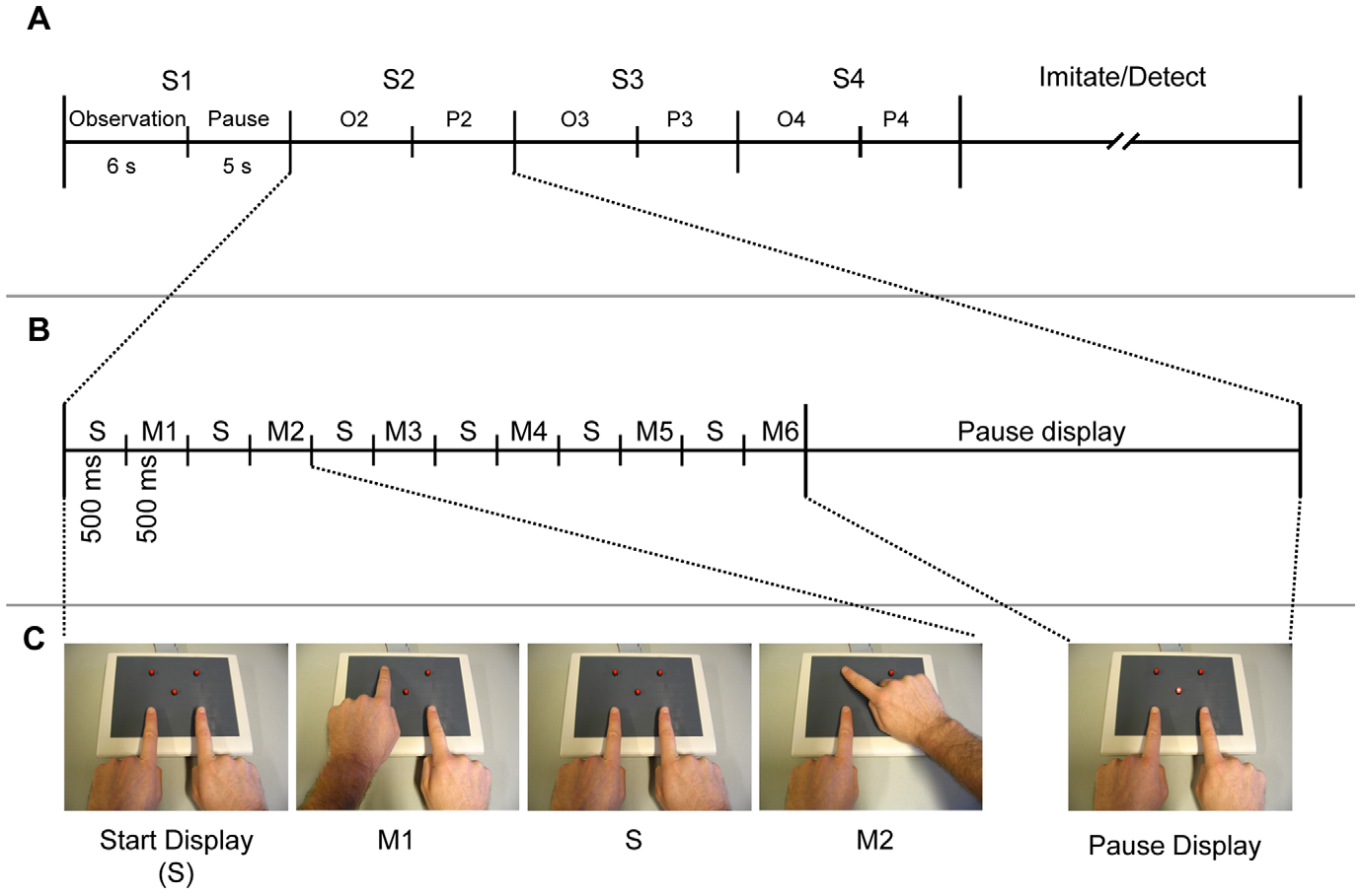
Schematic Overview of the Task Structure. (A) Each trial consisted of 4 repetitions (S1 – S4) of an observation interval (6 s) and subsequent pause interval (5 s). Following these 4 repetitions, subjects either tried to imitate the movement sequence from memory (imitation task) or responded if they had observed a catch movement in the action sequence (detection task). (B) Each observation epoch consisted of six button press movements, followed by a 5000 ms pause interval. Each button press movement consisted of a 500 ms display showing the left and right hand at the respective left and right start buttons (S) and a subsequent 500 ms apparent motion display (M) showing either the left or the right hand pressing an ipsi- or contralateral target button. (C) Examples of stimuli: Start display (S) showing the two hands pressing the start buttons; Example of an ipsilateral movement (M1); Example of a contralateral movement (M2); During the Pause display the central red button on the response box was illuminated.

The present study focuses on the learning of new action sequences through observation, analogue to the observations of etiologists and psychologists [1], [24]–[26] that new actions may be learned by recognizing the elementary motor acts and the specific order or sequence between them. For this type of observational learning to work, it is necessary that all movement elements are already within the motor repertoire of the observer. In the present study we used button presses that are well established in the motor repertoire of adult subjects and known for their ability to generate motor activation in an observer as shown by neuroimaging [27]–[29] and electro- and magnetoencephalographic studies [30]–[33]. Sequences of movements were repeated four times to allow subjects to effectively memorize the correct order of movements for subsequent reproduction (see Figure 1). Furthermore, each action sequence repetition was intervened by a short pause to study the neural processes supporting retention and rehearsal of these movements. That is, whereas much attention has been directed at action observation, an important requirement for successful imitation appears to lie in the ability of the observer to retain or rehearse the order of successive motor acts for subsequent imitation. Most likely, efficient rehearsal involves a process of mental imagery in which subjects mentally simulate the motor and perceptual effects that would accompany the actual execution of such actions [11], [34]. Consistent with this view, Buccino and colleagues [6] found that subjects activated large parts of the MNS, dorsal motor regions and the dorsolateral prefrontal cortex during a pause interval that intervened between the observation and execution phase in an experiment.

To capture the specific neural systems supporting imitation learning, a control task was included in which subjects viewed the same action sequences, without a requirement for subsequent reproduction, but had to subsequently report deviant movements.

## Results

The data of one of the participants contained too many artifacts to allow a reliable estimate of coherence. Data of this participant was excluded from both power and coherence analyses. Analysis of performance showed that the imitation task was challenging for most subjects. No participant succeeded in flawless performance. The amount of errors varied between 2 and 33 of the 40 trials between participants (mean percentage correct = 61.5%, SD = 23.3%).

### Power analysis

Statistical analysis of power in the alpha/mu frequency range was tested using a 2×2×2×4 within-subject ANOVA, with the factors Location (posterior electrodes pairs, central electrode pairs), Task (imitation, detection), Period (observation, pause), and Repetition (S1, S2, S3, S4). The percentage of correct sequence repetitions of each subject was added as a covariate to investigate if individual variations in alpha and mu power were systematically related to individual differences in imitation ability.

**Figure 2:**
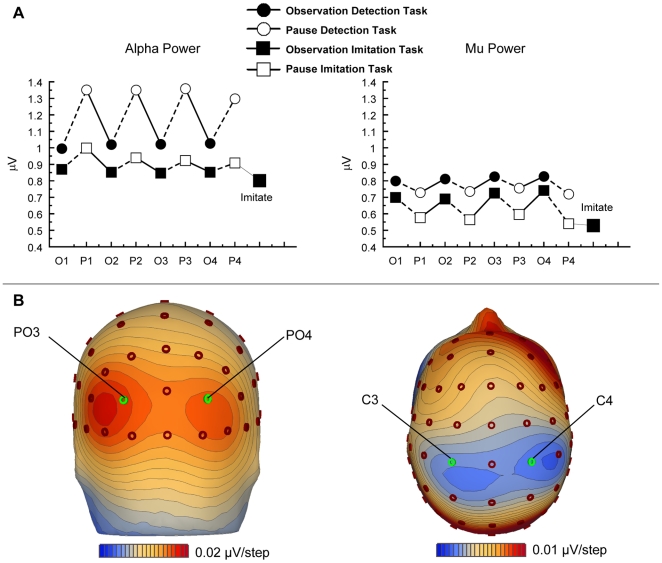
Effects on Alpha and Mu Power. (A) Alpha power (left) and mu power (right) during observation (O1, O2, O3, O4; black symbols) and pause (P1, P2, P3, P4; white symbols) intervals in the imitation task (squares) and the detection task (circles). Squares labelled with “Imitate” represent power levels during execution of the observed movement sequence. (B) Topographic representation of alpha power (left) maximal at bilateral parieto-occipital electrode sites, and mu power (right) overlying the left and right rolandic fissure. Alpha and mu topographies reflect the difference between pause and observation intervals in the detection task and the imitation task, respectively.

Figure 2 presents the grand average activity in the mu/alpha frequency range over central and posterior sites. Alpha power over extrastriate visual areas at electrodes PO3 and PO4 was found to be stronger than mu power over motor regions at electrodes C3 and C4, as reflected by an effect of Location, F(1, 13) = 21.6, p<.001. Furthermore, a main effect of Task was found, indicating that both mu and alpha were more strongly desynchronized in the imitation task than in the detection task, F(1, 13) = 18.8, p<.001. Mu suppression over central areas was stronger during pauses than during actual movement observation, while over posterior electrodes the reverse was found, i.e. alpha was suppressed more strongly during observation than pause intervals, reflected by an interaction between Location and Period, F(1, 13) = 29.6, p<.001. Importantly, the power increase in alpha over visual areas during the pause was stronger in the detection task than in the imitation task. On the contrary, over motor regions a stronger suppression of mu power was found during the pause of the imitation task as compared to the pause of the detection task, reflecting the interaction between Location, Task, and Period, F(1, 13) = 4.4, p = .056. This latter effect points to a functional difference between mu and alpha in their contribution to observation and retention of movements in the two tasks. In order to further specify this three-way interaction two post-hoc analyses were conducted focusing on posterior electrodes PO3/PO4 and central electrodes C3/C4, separately. The results of these analyses are presented hereafter.

The first ANOVA repeated measures contrast analysis directed at posterior alpha power revealed a main effect of Task reflecting stronger alpha desynchronization during the imitation task as compared to the detection task, F(1, 13) = 14.9, p<.01. In both tasks alpha desynchronized during observations of movements as compared to the subsequent pauses, as indicated by a main effect of Period, F(1, 13) = 11.2, p<.005. The topographical distribution of this effect is shown in the bottom left of Figure 2. Furthermore, a significant interaction between Task and Period was found, F(1, 13) = 9.3, p<.01, reflecting the stronger rebound effect during the pauses of the detection task than the imitation task. Other effects did not reach significance.

A second ANOVA repeated measures contrast analysis was directed at mu power over central electrodes. Effects in mu frequency power at electrodes C3/C4 are presented in the right panel of Figure 2. As on posterior electrodes, stronger overall desynchronization was found in the imitation task as compared to the detection task, reflected by a main effect of Task, F(1, 13) = 26.9, p<.001. Different from the stronger rebound in alpha power during pauses, mu activation was found to be suppressed more strongly during pauses than during observation intervals, as reflected by a main effect of Period, F(1, 13) = 6.3, p<.05. The topographical distribution of this effect is shown in the bottom right of Figure 2. A trend towards stronger desynchronization during pauses of the imitation task than in the detection task was found, as reflected by the interaction between Period and Task, F(1, 13) = 4.0 p = .066. Finally, an interaction between Period and Repetition was found, F(3, 39) = 4.6, p<.05. Subsequent contrast analysis to specify this interaction indicated that mu suppression during the last pause that preceded execution was stronger than in other pauses. In none of the analyses of power data did we find any effect of or interaction with imitation accuracy.

### Source localization of effects in mu and alpha power

**Figure 3:**
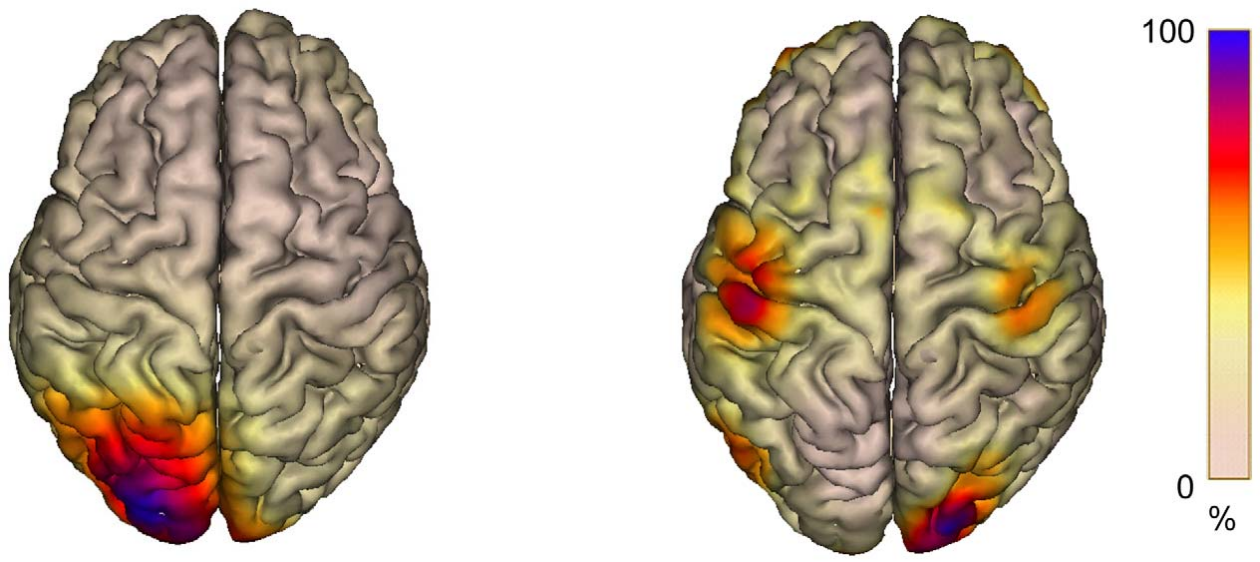
Localization of the Effects in Alpha and Mu Power. The alpha and mu effects (pause – observation) were calculated using minimum-norm analysis in BESA 5.1.6. (Brain Electrical Source Analysis, MEGIS Software, Germany). The minimal norm was computed with spatiotemporal weighting according to [49] fitted to an individual Talairach transformed brain surface. Effects are scaled with respect to the maximum difference between pause and observation intervals (100%, in blue). The left sources represent the alpha power, reflecting the difference between pause and observation intervals in the detection task, and the right sources represent the mu power, reflecting the difference between pause and observation in the imitation task.

Source localization of alpha power, i.e. the difference in power between pause and observation intervals in the detection task, showed maximal effects in the left occipital gyrus, extending to the parieto-occipital sulcus dorsally (see Figure 3 left). Mu power, reflected by the difference between pause and observation intervals in the imitation task, was localized to originate from the location of the hand area in the left and right central sulcus (see Figure 3, right). In addition, the minimum norm displays an effect in the right occipital gyrus reflecting the difference in alpha power between observation and pause intervals of the imitation task that was found concurrently over posterior areas.

### Coherence analysis

**Figure 4:**
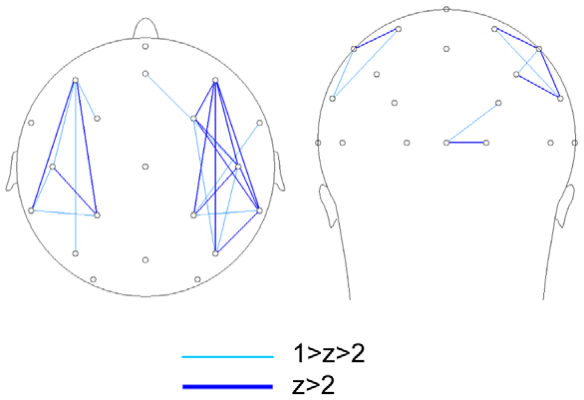
Fronto-parietal Coherence Network. Pairs of electrodes showing higher mu-frequency coherence during the observation of movement sequences than during the pause intervals. Only electrode pairs with the largest normalized coherence effects (1>z>2, z>2) are shown.

Figure 4 presents the network found to be activated more strongly during the observation of movements than during pause intervals (see Method section below for details on the procedure). This network basically consists of two individual networks each connecting parietal, central and frontal areas in each respective hemisphere.

**Figure 5:**
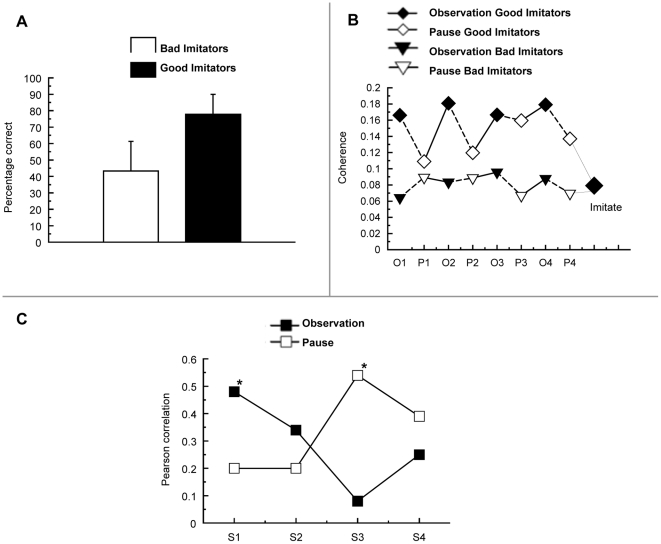
Interaction between Fronto-Parietal Coherence and Imitation Accuracy. (A) Median split between individuals with high accuracy (good imitators) and low accuracy (bad imitators). Values reflect the percentage of correctly imitated movement sequences. Error bars reflect the Standard Errors of Means. (B) Fronto-parietal coherence for good imitators (diamonds) and bad imitators (triangles) over time. Good imitators show stronger fronto-parietal coherence as compared to bad imitators. Squares labelled with “Imitate” represent coherence of bad and good imitators during execution of the observed movement sequence. (C) Pearson correlations between fronto-parietal coherence per sequence repetition (S1, S2, S3, S4) and interval (observation, pause) and imitation accuracy. The difference in performance between good and bad imitators is predicted by fronto-parietal coherence during the first sequence observation and the third pause interval for repetition. Significant correlations (p<.05) labelled with *.

An ANCOVA with Period (observation, pause) and Repetition (S1, S2, S3, S4) as within-subject factors and imitation accuracy as a covariate tested if coherence within the pair of fronto-parietal networks during observation for imitation was related to individual differences in imitation performance. Within the networks, coherence varied as a function of imitation accuracy, F(1, 12) = 4.8, p = .049, showing larger coherence for subjects with higher imitation accuracy. Furthermore, imitation accuracy significantly interacted with Period and Repetition, F(3, 36) = 4.0, p = .026. This interaction effect is reflected in Figure 5B where the coherence data is split between subjects with high and low imitation accuracy (Figure 5A). To further specify how coherence in the fronto-parietal networks is supporting imitation accuracy over time, a series of correlation analyses were performed between imitation accuracy and each consecutive observation and pause. One subject with exceptionally high coherence values (>0.6) was removed from the correlation analysis to avoid spurious correlations. This did not affect the overall pattern of correlation results. The results of this correlation analysis are represented in Figure 5C. Interestingly, a positive correlation was found between imitation accuracy and fronto-parietal coherence during the first observation of the sequence, r = 0.48, p = .05. No significant correlation was found in the subsequent pause, r = 0.20, p = .25. Interestingly, a reversed pattern of results was found during the third repetition, where imitation accuracy correlated positively with fronto-parietal coherence during the pause, r = 0.54, p = .027, but not during observation of movement sequences. These findings suggest that the critical difference between individuals who do well in imitation learning and those who do not, is that the former will i) coherently activate fronto-parietal systems during the initial observations of the movement sequence, and ii) are able to reproduce fronto-parietal activation during pause intervals after having observed several repetitions of the same movement sequence.

## Discussion

In the present study we investigated the neural mechanisms supporting the acquisition of new action sequences focusing on brain oscillations in the mu/alpha frequency range. To determine the functional contribution of mu and alpha rhythms in observation learning and the possible coupling between mu and alpha generators in this process, both power and coherence analysis were conducted. Results point towards the independency of mu and alpha generators in observation learning as reflected by changes in mu and alpha power over time and the absence of coherence between parieto-occipital and central electrodes. Mu desynchronization was found maximal during pause intervals, consistent with the hypothesized rehearsal of movement sequences via motor imagery. Alpha desynchronization, instead, was stronger during the observation of action sequences, reflecting involvement in perceptual processing of action sequences. These findings argue against the global entrainment hypothesis [15] which proposed functional coupling between alpha and mu generators as the basic psychophysiological mechanism supporting imitation learning. Instead, results provide clear evidence for the alternative, i.e. the direct matching hypothesis of observational learning [1] which proposed that imitation learning relies on motor resonance in the MNS, independent from visual processing. This hypothesis receives support from the discovery of two ipsilateral parieto-frontal networks overlying the two respective hemispheres that were found activated during observation of action sequences for imitation. Importantly, individual differences in the strength of coherence in these two parallel networks were found to predict imitation accuracy, underlining their central role in observational learning.

Previous studies on motor resonance during action observation typically focused on the mu rhythm and systematically ignored possible confounding modulations in alpha over posterior regions. In the current study this possible confound was investigated by studying discrepancies between alpha and mu power during the different phases of imitation learning. Consistent with the prediction from the global entrainment hypothesis that imitation learning would be supported by both visual and motor processes [15] alpha and mu rhythms were both found to desynchronize when participants observed and remembered sequences of movements for subsequent imitation, as compared to a control task in which subjects detected deviating (thumb) movements. This suggests that both visual and motor processes contributed to the current task of observational learning. Importantly however, no functional coherence was detected between visual and motor processes within the alpha/mu band as was predicted by the global entrainment hypothesis [15] and modulations in alpha and mu power were found to be largely independent from each other, arguing against the functional integration of alpha and mu oscillations in support of action observation and observational learning.

The independence between visual and motor oscillations in the mu/alpha range differs from other studies that found posterior – central coherence in the 8–13 Hz frequency range [35], [36]. In an experiment by Classen and others [36], for instance, subjects had to exert an oscillating force with their right index finger on a strain gauge. It was shown that central-posterior alpha coherence was highest when the force exerted had to be synchronized with a visually presented oscillating target dot. Another example is provided by Hummel and Gerloff [35] who simultaneously presented a tactile Braille letter to the right index finger tip and a visual Braille letter on a computer screen, resulting in a phasic increase in alpha coherence between central and posterior electrodes that was higher if subjects were successful in verifying if the visual and tactile letter matched or not. The main difference between these studies and the current study is probably that task properties of these other studies demand the immediate integration between visual and sensorimotor information, whereas for observational learning the requirement for temporal integration between visual and motor information is less demanding. One other possibility may be that for observational learning to be effective, motor representations are to be protected from visual input that may interfere with the retention or rehearsal of action sequences, as was indicated by behavioral studies that investigated interference effects during learning of whole body movement patterns [37]. Irrespective, however, of the precise reason for the absence of visuomotor integration during imitation learning, the coherence data of the current experiment suggest that such an integration of visual and sensorimotor information is not a necessary requirement for observational learning to take place.

In line with the conclusion that visual and motor processes operated largely independently from each other, several dissociations between visual and motor processes as reflected in alpha and mu power were found. One prominent difference in mu and alpha power was that alpha desynchronization was strongest during the observation interval, suggesting maximal sensitivity to visual input, whereas mu desynchronization was strongest during the pause intervals, suggesting active involvement in the rehearsal of action sequences for subsequent reproduction. The latter finding of enhanced mu desynchronization during pause intervals is particularly interesting because it suggests that subjects engaged in active motor imagery supporting the maintenance and/or encoding of action sequences in memory for subsequent reproduction. Consistent with this interpretation, desynchronization effects in mu power were found to be stronger in the imitation task as compared to the detection task. Source localization of mu desynchronization using a minimum norm approach showed activation to originate from pre- and post-central gyri around the location of the hand area in the left and right central sulcus. Consistent with the interpretation that mu desynchronization during pauses may be a measure of motor imagery, EEG studies have reliably found motor imagery accompanied by desynchronization in mu oscillations originating from primary sensorimotor areas [38], [39].

Previous studies investigating action observation of finger movements, facial expressions and goal directed grasping [17], [18], [40] have firmly implicated mu desynchronization as a measure of motor resonance. This view has furthermore been strengthened by studies that found stronger mu desynchronization to the observation of actions that were already established in the observer's motor repertoire (e.g. dancing moves by professional dancers [9], or infants' ability to crawl or walk [10]) as compared to actions that were not mastered yet. The present findings further extend the functional role of mu desynchronization beyond action observation by showing that mu is not only involved in the observation of already learned actions but also directly supports the acquisition of new actions. Importantly, although finger movements are probably well established in the observers' motor repertoire, covert motor activation was stronger during the pause intervals than during actual action observation. This result points towards the involvement of mu in both action observation and motor imagery, and suggests that, when studying imitation and learning of new motor repertoire, one should be careful to distinguish between motor activation resulting from action observation and motor activation reflecting motor imagery.

Consistent with the direct matching hypothesis, coherence analysis rendered two ipsilateral fronto-parietal networks, one over the left and one over the right hemisphere, revealing stronger fronto-parietal coherence during the observation of movement sequences than during subsequent pauses. The properties of these two networks, i.e. their fronto-parietal distribution, their ipsilateral organization and their sensitivity to the observation of movements, matches closely with the known properties of the MNS as uncovered by anatomical and electrophysiological studies in the macaque brain [41]. These studies have found strong parallel and reciprocal connections between ipsilateral regions of the frontal and parietal lobes supporting specific sensorimotor transformations for action execution and action observation [42]. The present study is the first to find a possible correlate of the MNS reflected in fronto-parietal coherence. These findings call for further confirmation, e.g. by studies that combine EEG and fMRI.

Importantly, the strength of fronto-parietal coherence during imitation learning was found to be positively related to the accuracy of subsequent imitation. This suggests that the strength of the functional coupling between parietal and frontal parts of the network for imitation learning is decisive for the accuracy of newly acquired action sequences. Neuroimaging studies have suggested different functional roles for frontal and parietal parts of the MNS [43], [44], for processing the goals of actions (i.e. the end-location or the object being targeted) and the means of observed actions (i.e. the selected grasp or movement trajectory). In the present sequence learning task participants needed to integrate information about the hand that was used (action means) and the specific target button visited (action goal) for each consecutive pointing action. A reasonable inference is that enhanced coherence between frontal and parietal MNS regions may support the integration of information about action goals and means during action observation and rehearsal for subsequent imitation. Consistent with this interpretation, Fogassi et al [45] found that the discharge rates of cells supporting the execution and observation of grasping acts in the macaque inferior parietal lobe were sensitive to the final goals of grasping actions (i.e. grasping to place vs. to eat), i.e. reflecting the capacity of the MNS to anticipate and integrate successive motor acts (grasping and placing) in an action chain. Interestingly, the anticipatory effects of action chaining were found both during execution and observation of similar grasping acts by an experimenter, confirming the functional contribution of the MNS to both action production and action observation.

Regarding the present study, correlation analyses between imitation accuracy and fronto-parietal coherence per repetition and period, indicated that performance differences were most clearly expressed during the first observation of the movement sequence and the third pause interval. This pattern suggests that, in addition to the general difference in fronto-parietal coherence between good and bad imitators, good imitators pay better attention to, or are better able to integrate information, about action goals (left/right button) and action means (left/right hand) during the first sequence presentation. Furthermore, the correlation during the third pause interval suggests that the functional contribution of fronto-parietal coherence is not limited to action observation only, but may also support the active rehearsal of a movement sequence in memory [45]. The positive correlation between imitation accuracy and fronto-parietal coherence during the third pause interval suggests that good imitators were better able to activate a correct representation of consecutive goal-means relations in memory for subsequent reproduction.

An interesting dissociation is that imitation accuracy correlated with fronto-parietal coherence, but not with mu and alpha power. The absence of a correlation between imitation accuracy and power measures confirms the interpretation that both alpha and mu power represent activation in relative peripheral systems, i.e visual extrastriate regions and sensorimotor cortex, that may not be directly involved in representing the order of an action sequence or the integration between goals and means, but seem to provide low level support during the observation and motor imagery of action sequences in memory. Instead, both the topography of fronto-parietal coherence overlying higher-order motor systems in frontal and parietal cortices and the relation between coherence strength and imitation accuracy point towards a central role in imitation learning.

An important question to ask is to what extent the fronto-parietal coherence that we found to support observational learning is representative for the learning of new motor repertoire in more naturalistic settings. One property of the present task is that was quite difficult for subjects to accomplish faultless imitation of the entire sequence. The complexity and difficulty of the present sequence learning task may not, however, be that different from natural conditions in which individuals e.g. have to learn a new sequence of dance steps, or learn the correct order of actions (switching gears, use indicator, looking, etc.) when approaching a crossroad during driving lessons. It is still unclear however to what extent fronto-parietal coherence may support other types of observational learning where the focus is not on combining established movement elements in a new arrangement, but on the learning of an entirely new movement primitive, for instance when children in elementary school have to imitate a teacher's posture of holding a pen to allow writing.

In conclusion, the present study was successful in uncovering different neural mechanisms and their potential roles in observational learning. Observational learning was found to be supported by a pair of fronto-parietal coherence networks, one overlaying the left and one overlaying the right hemisphere. Coherence in these fronto-parietal networks likely provides a direct measure of activation and coherence within the human MNS. Fronto-parietal coherence predicted the accuracy of subsequent imitation, indicating a central role for these structures in imitation learning. In addition, observational learning was found to be supported by activation in visual extrastriate and central motor regions, reflected by power modulations in alpha and mu components. Although visual and motor processes reflected in alpha and mu power may be jointly activated in support of action observation and motor imagery, both the variance in mu and alpha power over time and the absence of coherence between occipito-parietal and central motor regions argue against the global entrainment hypothesis and the hypothesized coupling between alpha and mu oscillations in support of observational learning. Although the present study indicates a role for visual processes in imitation learning, the current findings generally confirm the direct-matching hypothesis of imitation learning which states that observational learning depends on motor resonance in high-level motor structures making up the core MNS. In addition the results obtained during the pause intervals make clear that imitation learning, for a good part, relies on motor imagery and that studies investigating observational learning will need to distinguish between motor activation resulting from action observation and motor imagery for subsequent imitation. We hope that the current study will inspire other researchers to further investigate the neural and functional basis of one of most central of human abilities, i.e. the capacity to learn new behavioral repertoire, through action observation.

## Materials and Methods

### Participants

The study was approved by the ethics committee of the Faculty of Behavioural Sciences from the University of Twente. Fifteen university students (9 females) participated in the experiment and were given course credits as reward. All participants were between 18 and 26 years of age, with a mean age of 20.5 years (SD = 2y). Subjects were right handed as assessed by the Annett Handedness Inventory [46] and provided written informed consent before the study.

### Apparatus

A custom made response-box was used, consisting of four buttons that were arranged in a square shape and a fifth button located in the middle (see Figure 1). Each button had a built-in red LED which could be illuminated. The response-box was connected to a PC and positioned in front of a 17 inch computer monitor (Philips 107T5, refresh-rate 85 Hz) that was used for presenting visual stimuli to subjects. The experiment was programmed and controlled in E-prime (Version 1.2., Psychology Software Tools, Inc. Pittsburgh, USA).

### Procedure

In each trial, participants were presented with a sequence of 6 button presses on the computer screen. For each button press movement two subsequent pictures were presented, the first one showing the index fingers of both hands at the left and right (proximal) starting buttons of the response box, and a second picture showing one of the hands pressing one of two (distal) target button (see Figure 1). Six individual button press movements were presented in succession with each movement lasting 1000 ms (12 pictures of 500 ms each). The end-locations of each movement were selected from a total of four pictures that reflected the different combinations of hand (left, right) and button (left, right). Each of these four movements was presented at least once in every sequence of movements, but never thrice, and the same movement was never repeated more than once in succession. Directly following the sequence of six movements, a 5000 ms pause was administered during which a picture was presented showing the hands in the starting posture and the central red button illuminated to demarcate the pause interval from the sequence repetitions.

In the imitation task subjects were asked to replicate the observed sequence of movements after having seen 4 sequence repetitions, using the same response box that was depicted on the computer screen. Subjects first pushed the two buttons that represented the starting positions. As a result, the central button on the response pad was illuminated for 5000 ms, during which a text was presented on the screen informing the subject that, as soon as the centre button on the response pad was turned off, they had to execute the observed movement sequence as accurately as possible. Accuracy of each movement was evaluated with respect to whether subjects released the correct starting button (i.e. whether they used the correct hand) and whether they pushed the correct target button. If either the wrong hand was used or the wrong target button was pushed in one of the movements, subjects received feedback that they made one or more errors. If the sequence of movements was executed correctly, the time to execute the entire sequence was presented on the screen. Subjects pushed a button on the button pad when they were ready to start a new sequence.

In the detection task, the movements and sequences were the same as in the imitation task with the exception that in 20% of the presented sequences one of the movements was replaced by a catch movement in which a target button was pushed with the thumb instead of the index finger. After each presentation of the sequence, subjects had to report if a catch movement had occurred. If so, they had to push any button on the response-box within the 5000 ms during which the visual pause was administered. If the subject missed a catch movement or responded falsely, i.e. when no deviant was shown, “WRONG!” was projected over the warning display for 1000 ms, after which a new, neutral warning signal was displayed for 5 s, and the experiment continued. Sequences containing catch movements or false alarms were omitted from the EEG analyses to rule out the possibility of response preparation contaminating the detection task.

### Electrophysiological recording

Sixty-one-channel EEG was recorded according to the International 10–20 System of Electrode Placement [47], using a BrainVision QuickAmp amplifier (Brain Products GmbH, Munich, Germany) in combination with shielded Ag/AgCl electrodes. Electrode impedances were kept below 10 kΩ. EEG was sampled at 500 Hz with a 140 Hz low pass filter and a 50 Hz notch filter. An average reference was used during acquisition of the EEG signal.

Because arm and hand movements are known to influence the mu rhythm, participants were instructed to position their hands along each side of the response box on the table and to move as little as possible during sequence observation in both tasks. Electromyograms (EMG) were measured using bipolar electrodes over *flexor carpi radialis* and *extensor pollicis longus* on both forearms, so that covert movements made by participants could be detected [17]. Vertical and horizontal electro-oculograms (EOG) were recorded using bipolar electrodes placed at the supraorbital and infraorbital ridge of the right eye and the outer canthi of the left and right eye respectively. Participants were instructed to fixate on the middle button of the response box presented on the computer monitor during sequence observation in both tasks. EEG, EMG and EOG data were analyzed offline using BrainVision Analyzer v1.05 (Brain Products GmbH, Munich, Germany) and BESA 5.1.6. (Megis Software GmbH, Gräfelfing, Germany).

### Analysis

#### Power analysis

EEG recordings in the imitation and detection tasks were analyzed separately for observation intervals and pause intervals to distinguish between activation generated by the observation and retention of movements. An additional analysis was directed at the execution interval of the imitation task during which participants performed the movement sequence from memory. Observation intervals (6000 ms interval consisting of 6 consecutive button presses of 1 s each), pause segments (5000 ms interval following each sequence repetition), and the execution interval (movement onset until movement end), were segmented into equally sized epochs (of 512 time points). Epochs containing EEG artefacts or EMG activity (execution interval excluded) were discarded from analysis using an automated procedure. This resulted in a rejection of 15.5% of all epochs in the detection task and 8.8% of all epochs in the imitation task. Furthermore, segments in which eye blinks were detected were discarded, resulting in an additional rejection of 10.4% of all trials of the detection task and 14.9% of the imitation task. For each individual subject, Fast Fourier Transforms (FFT) were performed on the artefact free EEG segments (512 points, Hanning window, 10% envelope), and averaged to create separate power frequency spectra for the observation and pause intervals per repetition in the imitation and detection tasks.

Each participant's individual mu rhythm band was determined by subtracting the power frequency spectrum of the sequence execution phase during the imitation task (which contained the lowest mu activation due to hand movements) from the power frequency spectrum during the observation phase of the detection task (which contained the highest mu activation). For each participant this difference was topographically mapped and the location of maximal mu modulation identified to determine each participant's individual mu frequency [48]. Mu power was calculated using a +/−0.5 Hz frequency bandwidth around each individual's mu frequency. Similarly, for each participant the individual alpha rhythm was determined by subtracting the frequency spectrum in the condition with the lowest alpha power (observation of movements in the imitation task) from the condition with the highest power of alpha (pause in the detection task). For each individual, the posterior location and the frequency at which alpha power was maximal were identified. Alpha power was calculated using a +/−0.5 Hz bandwidth around the each individual's alpha frequency. For statistical analysis bilateral electrodes were identified at which modulations in mu power (C3 and C4) and alpha power (PO3 and PO4) were found maximal over subjects. Data from left and right hemispheres were pooled in the statistical analyses.

#### Source localization

Localization of the effects in alpha and mu power (pause-observation) was established using minimum-norm analysis in BESA 5.1.6. (Brain Electrical Source Analysis, MEGIS Software, Germany). The minimal norm was computed with spatiotemporal weighting according to [49] fitted to an individual Talairach transformed brain surface.

#### Coherence analysis

Raw data were filtered off-line (1–50 Hz, slope 12 dB/octave) in advance of coherence analysis. Epochs containing artefacts were discarded from analysis using an automatic procedure. Segments with eye blinks were rejected. Before coherence analysis a current source density analysis was conducted (order of splines = 4, 10 polynomials) to remove spreading of nearby source activation to neighbouring electrodes [50], [51]. An FFT was performed on each segment before calculating the coherences within each segment. Coherence values between electrode pairs were calculated for the frequencies within the 8–13 Hz frequency range. Similar to the analysis of power, each subject's individual coherence frequency was identified by averaging coherence across all electrode pairs and tasks and determining the peak frequency with maximum coherence. Individual coherence values were calculated by taking the average coherence activation in the +/−0.5 Hz band encompassing each individual's coherence peak frequency.

The aim of the coherence analysis was to identify functional networks differentiating between the observation and retention of movements for imitation. To identify the networks that show differential coherence between movement observation and subsequent pauses, coherence averages of the observation interval were subtracted from coherence averages during the pause. These coherence difference scores were transformed to z-values to identify electrode pairs with strongest coherence during the observation of movements (z-values smaller than -1) and electrode pairs with strong coherence during pause intervals (with z-values larger than 1). Inspection of the resulting networks indicated a specific pair of fronto-parietal networks that were maximally coherent during the observation of movements. Statistical analysis of coherence in the observation network was analyzed using a within-subjects ANOVA repeated measures analysis to determine effects of Period (pause, observation) and Repetition (S1, S2, S3, S4), with imitation accuracy as a covariate to investigate associations with individual differences in imitation ability.
